# Bidimensional Spectroelectrochemistry with Tunable Thin-Layer Thickness

**DOI:** 10.1021/acs.analchem.4c01132

**Published:** 2024-05-30

**Authors:** Martin Perez-Estebanez, Juan V. Perales-Rondon, Sheila Hernandez, Aranzazu Heras, Alvaro Colina

**Affiliations:** † Department of Chemistry, Universidad de Burgos, Pza. Misael Bañuelos s/n, E-09001 Burgos, Spain; ‡ Hydrogen and Power-to-X Department, Iberian Centre for Research in Energy Storage, Polígono 13, Parcela 31, ≪El Cuartillo≫, E-10004 Cáceres, Spain; § Chair of Analytical Chemistry II, Faculty of Chemistry and Biochemistry, Ruhr University Bochum, Bochum 44801, Germany

## Abstract

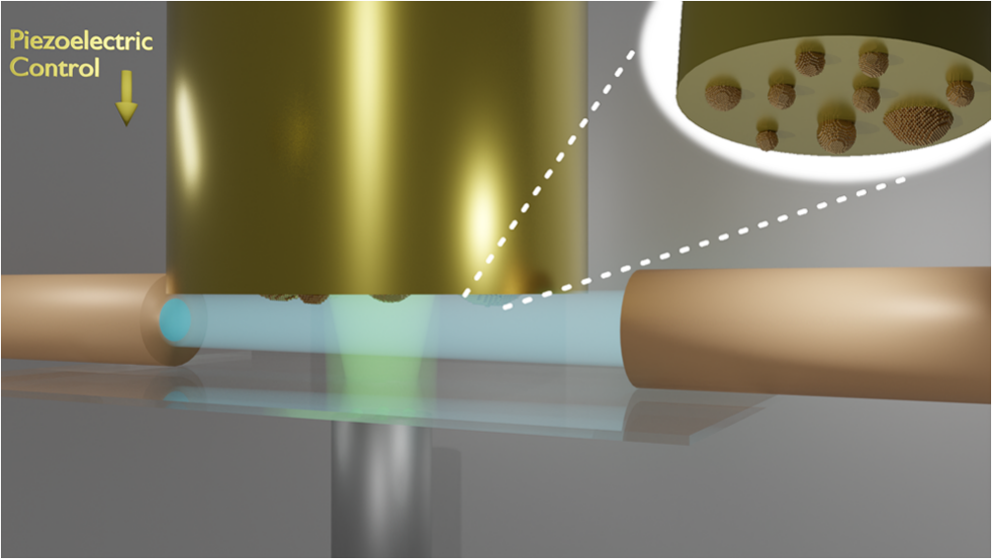

Bidimensional spectroelectrochemistry (Bidim-SEC) is an instrumental technique that provides *operando* UV/vis absorption information on electrochemical processes from two different points of view, using concomitantly a parallel and a normal optical configuration. The parallel configuration provides information about chemical species present in the diffusion layer, meanwhile the normal arrangement supplies information about changes occurring both in the diffusion layer and, mainly, on the electrode surface. The choice of a suitable cell to perform Bidim-SEC experiments is critical, especially while working under a thin-layer regime. So far, most of the proposed Bidim-SEC cells rely on the use of spacers to define the thin-layer thickness, which leads to working with constant thickness values. Herein, we propose a novel Bidim-SEC cell that enables easy-to-use micrometric control of the thin-layer thickness using a piezoelectric positioner. This device can be used for the study of complex interfacial systems and also to easily measure the key parameters of an electrochemical process. As a proof of concept, the study of the roughening of a gold electrode in KCl medium is performed, identifying key steps in the passivation and nanoparticle generation on the gold surface.

Spectroelectrochemistry (SEC) involves a set of techniques, combining an electrochemical technique with a spectroscopic one, providing *operando* information about the electrochemical process.^1−4^ Among all of the possible spectroscopic techniques used in SEC, UV/vis absorption SEC (UV/vis-SEC) has been widely used, thanks to its versatility, allowing us to study the redox properties of different molecules,^5,6^ to elucidate the reaction mechanisms,^7^ or to perform quantitative analysis.^8−10^ Recently, UV/vis-SEC has been reported for the characterization of complex systems such as energy storage devices.^11^

UV/vis-SEC can be carried out both in semi-infinite and in thin-layer diffusion regimes,^12−15^ with the latter being one of the most widely used approaches. Nevertheless, thin-layer measurements present two fundamental drawbacks. On the one hand, thin-layer thickness control can be complex and often requires the use of spacers, which limit its fine control.^16,17^ On the other hand, this configuration usually involves high ohmic drops.^18−20^ This makes the design of the cell critical for SEC experiments. Some authors have reported a remarkable drift of the spectral baseline depending on the cell used,^21^ which makes it more difficult to obtain reliable data, and although such drifts can be corrected with strategies such as taking derivative measurements,^22−24^ it is certainly ideal to use optimum cell designs to avoid such baseline changes.

Over the years, numerous approaches to design thin-layer SEC cells have been proposed.^13,20,25−29^ One of the most widely used cells in UV/vis-SEC has been the one proposed by Heineman et al.,^18,30^ in which the solution is placed in the optical pathway defined by an optically transparent electrode (OTE) and a transparent inert wall. Other strategies developed for SEC cells are based on thin spectrophotometric cuvettes,^31,32^ in which a platinum mesh or an indium tin oxide (ITO) electrode is usually selected as the OTE. An alternative to the usage of platinum meshes consists of utilizing the so-called honeycomb electrodes.^33^ However, although these electrodes have a good SEC response, a very complex diffusion regime is generated due to the geometry of the electrodes,^33^ which makes the extraction of quantitative information more challenging.

To improve the sensitivity in thin-layer experiments, devices based on disk electrodes have also been developed, using an inert wall and thin spacers to define the space where the solution is confined.^16,17,34^ The latter allows us to work in an optical configuration parallel to the electrode, which maximizes the optical pathway and thus the spectroscopic response. Recently, simpler cells using screen-printed electrodes (SPEs) combined with small-diameter optical fibers have been proposed for SEC. This new assembly allows defining a thin layer with a thickness value equal to the size of the optical fibers by placing an inert wall that confines the solution.^13^ Nevertheless, the use of optical fibers introduces new problems due to their cladding, which is usually around 30 μm in thickness, hindering the interrogation of the 30 μm of solution closest to the electrode. This blind spot in the parallel configuration can hinder the study of processes taking place in the vicinity (a few micrometers) of the electrode surface.

In the present work, a new device to carry out UV/vis-SEC studies in a thin-layer configuration is proposed. In this new approach, the working electrode (WE), controlled by a piezoelectric positioner, is placed between two optical fibers, which are fixed to a quartz plate. Thus, by controlling the position of the WE in the solution between the two bare optical fibers, it is possible to sample the first micrometers of solution adjacent to the electrode in the parallel direction. Simultaneously, a reflection probe is perpendicularly focused on the electrode surface, carrying out measurements in a normal configuration. Using the proposed device, bidimensional UV/vis-SEC (Bidim-SEC) measurements can be easily performed simultaneously by combining UV/vis-SEC in parallel and normal configurations with a tunable thickness of the thin layer.

To demonstrate the correct performance of the Bidim-SEC cell, SEC experiments with *o*-tolidine (*o*-Tol) were carried out to validate the Bidim-SEC device. Finally, to exploit its usefulness in the study of complex reaction mechanisms, this new SEC cell was employed to study the generation of Au nanoparticles (AuNPs) on a Au disk electrode by using oxidation–reduction cycles (ORC), a classical strategy for the generation of *surface-enhanced Raman scattering* (SERS) substrates.^36−38^

## Experimental Section

### UV/vis Bidimensional Spectroelectrochemistry

Bidim-SEC measurements were simultaneously performed using two different optical configurations: normal and parallel with respect to the electrode surface. In the normal arrangement, the incident light beam is perpendicular to the WE surface, providing information about both the species in the diffusion layer and those adsorbed/deposited on the electrode surface; whereas in the parallel arrangement, the light beam passes parallel and adjacent to the electrode surface, providing only information about soluble compounds in the first microns of solution interrogated by the light beam.^39^

In the normal configuration, a reflection probe was used to interrogate the solution and the electrode surface. In the parallel configuration, two bare optical fibers (100 μm diameter, Avantes) were fixed and aligned with a quartz crystal (4 cm × 1.2 cm × 0.12 cm). For each configuration, the acquisition time was selected to ensure enough light intensity along the full spectrum, with the typical acquisition times in the range of 100 ms for the normal configuration and 100–1000 ms for the parallel configuration. Larger acquisition times were used in the parallel configuration for experiments with the thinnest layer thicknesses. Full experimental details are provided in the Supporting Information (SI).

### Electrochemical Setup

A 2.9 mm diameter Au rod (Goodfellow, 99.5 %) embedded in Teflon was used as the WE. The WE was polished with 0.05 μm alumina to obtain a mirror-like surface and sonicated for around 3 min in Milli-Q water before each experiment. A gold strip (40 mm × 2 mm) was used as the counterelectrode (CE) which was wrapped around a homemade Ag/AgCl (3 M KCl) reference electrode (RE). All potentials were referenced to 3 M Ag/AgCl.

### Description of the UV/vis Bidimensional SEC Cell

**Figure 1:**
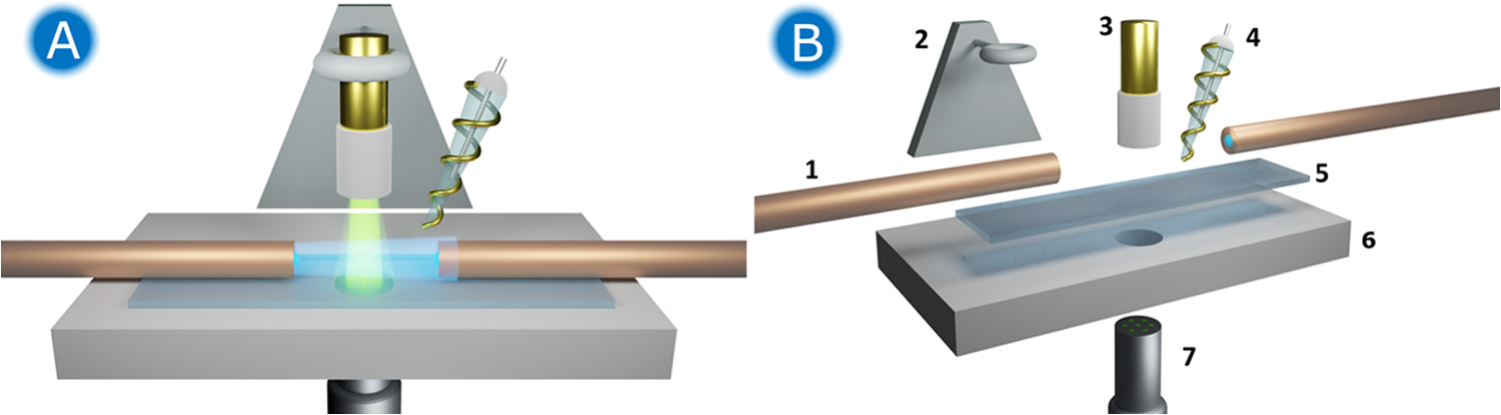
(A) Schematic of the proposed Bidim-SEC cell. (B) Itemization of the components of the cell: (1) emission and reception optical fibers used in the parallel configuration; (2) micrometric positioner; (3) Au-WE; (4) 3 M Ag/AgCl RE and Au strip CE; (5) quartz slide; (6) Teflon plate substrate; (7) reflection probe used in the normal configuration.

A schematic of the proposed thin-layer Bidim-SEC cell is presented in Figure 1. Figure 1A provides a detailed view of the distribution of the elements in the cell. For normal configuration SEC, a reflection probe (7) was embedded in a Teflon plate substrate (6) and placed under the WE (3). A 1.2 mm thick quartz slide (5) was used both as an inert wall to generate a thin layer and to separate the reflection probe from the WE at a suitable distance to obtain a suitable optical response. It should be noted that the optical probe has to be in contact with the quartz crystal to avoid any reflection that yields parasite radiation. For the parallel configuration, two 100 μm bare optical fibers (1) were aligned with and fixed on the quartz crystal. The z-position of the WE was controlled by a micrometric positioner (2, Newport M460P series). The thickness of the thin layer was defined by the distance between the WE and the quartz crystal. Figure 1B lists the components of the Bidim-SEC cell.

The WE was initially placed above the optical system, vertically aligned with the reflection probe. Separation of the optical fibers greater than the diameter of the WE allowed placing it between the optical fibers and sampling all of the solution close to the electrode. Before starting the experiment, the WE was placed between two bare optical fibers (Figure 1B) using a piezoelectric positioner. To define the thickness of the thin layer, a first approximation of the electrode to the glass slide was carried out. The contact of the WE and the quartz crystal was ensured with the aid of a video macrolens camera (SONY, TW-CL160), setting that position as 0 μm distance. After that, the WE was retracted up to the desired thickness of the thin layer (defined precisely by using a piezoelectric positioner). Once this distance was defined, an aliquot of the desired solution was added to the system, ensuring good electrical contact between the three electrodes and a good immersion of the bare optical fibers in the solution. The RE was placed as close as possible to the WE to minimize the ohmic drop, Figure 1B. The CE was wrapped around the RE.

With the instrumental approach proposed in this work, a thin-layer cell with a variable thickness can be easily obtained. The minimum value of the thickness of the thin layer that can be set in this setup was 30 μm. At smaller thicknesses, the light intensity that reaches the detector is very low since the WE blocks most of the light in the optical pathway, resulting in a poor signal-to-noise ratio.

## Results and Discussion

### Validation of the UV/Vis Bidimensional Spectroelectrochemical Cell

**Figure 2:**
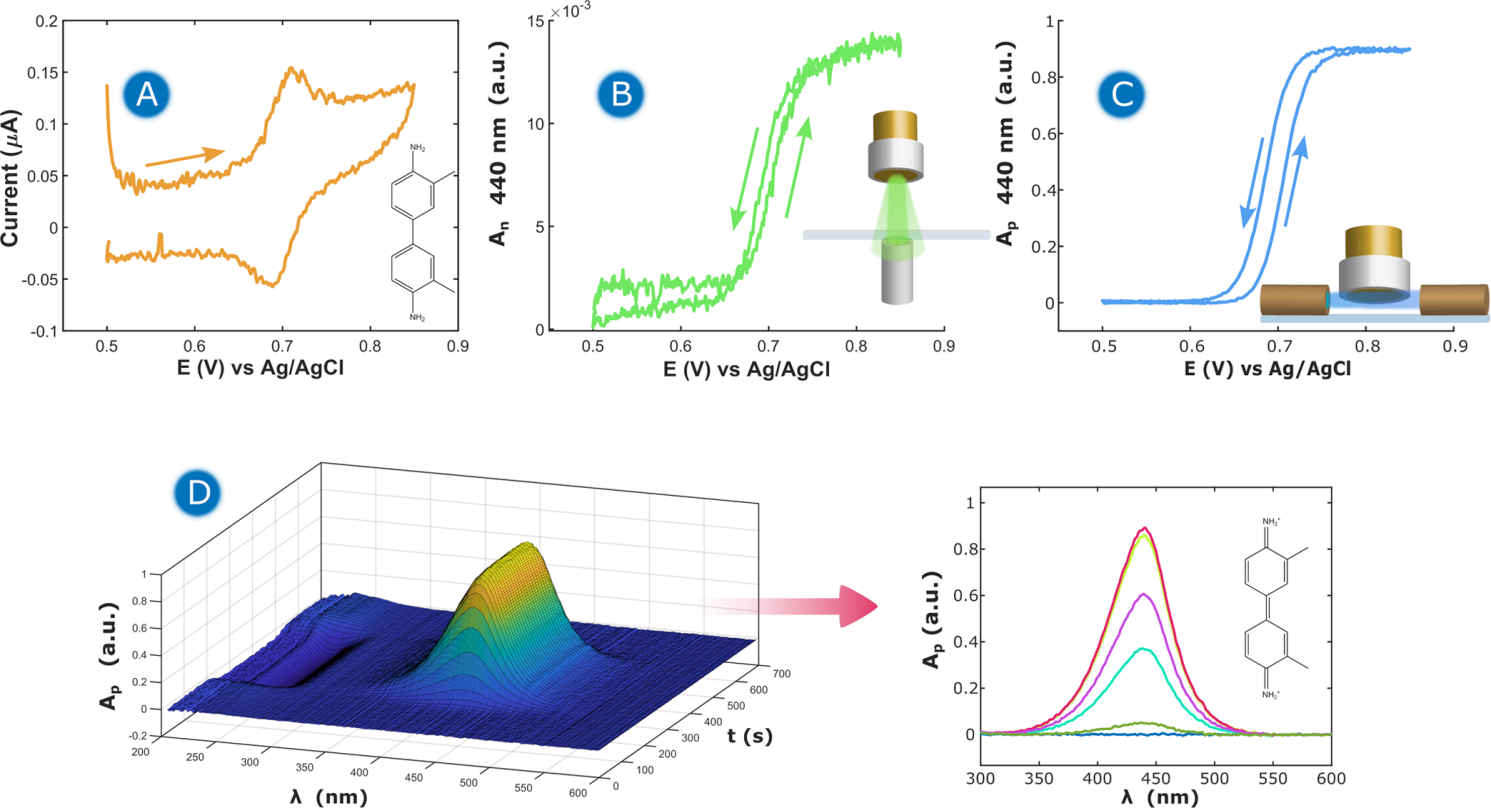
Bidimensional SEC responses of 0.05 mM *o*-Tol + 1 M HClO_4_ + 0.5 M acetic acid on a Au rod WE working in a thin-layer regime. (A) CV response of *o*-Tol reversible oxidation. CVA at 440 nm in (B) normal and (C) parallel configurations. (D) Surface plot of the evolution of the UV/vis absorption spectra with the potential applied, recorded in the parallel configuration. Thin-layer thickness: 50 μm. Scan rate: 1 mV s^–1^.

A typical molecule used to validate UV/vis-SEC devices, *o*-tolidine (*o*-Tol),^14,16,18,40^ was selected to demonstrate the good performance of the proposed Bidim-SEC cell. *o*-Tol does not absorb light in the visible region; however, *o*-Tol can be oxidized in acidic medium to yield the *o*-tolidinium (*o*-Tol^+^) cation, which shows a strong absorption band at 440 nm. UV/vis absorption spectra of the reduced and oxidized forms of *o*-Tol are significantly different, making it possible to follow the electrochemical changes of the system by studying the spectroscopic response, as shown in Figure 2.

Cyclic voltammetry (CV) was performed to observe the redox behavior of *o*-Tol in 1 M HClO_4_ + 0.5 M acetic acid medium. For this experiment, the thickness of the thin layer was fixed to 50 μm. Figure 2A shows the well-defined CV response obtained during the experiment, showing a peak-to-peak separation of 20 mV. This small hysteresis is close to the zero value expected in a thin-layer experiment.^16,40^

The recorded optical responses are shown in Figure 2B–D. Figure 2D shows a surface plot describing the evolution of the full UV/vis absorption spectra recorded in the parallel configuration during the experiment. It is noteworthy that an absorption band evolves around 440 nm, which corresponds to the electrogeneration of the *o*-Tol^+^ cation, as a result of the oxidation of *o*-Tol. The cyclic voltabsorptogram (CVA) or evolution of the absorbance at 440 nm during the experiment is presented in Figure 2B,C in normal and parallel configurations, respectively. The CVAs in the two configurations allow us to clearly follow the evolution of the electrochemical processes.

As shown in Figure 2B,C, in the two configurations, an increment of absorbance at this wavelength and at potentials above +0.65 V is observed, due to the oxidation of *o*-Tol to the *o*-Tol^+^ cation. As is expected, absorbance values in the parallel configuration are higher than the ones in the normal configuration, due to the difference in the optical path length, which is 2 times the thickness (*d*) of the thin layer (*d* = 50 μm in this case) in the normal configuration and 2.9 mm (diameter of the WE) in the parallel configuration.

The CVAs shown in Figure 2B,C demonstrate the reversibility of the redox process since in both configurations the absorbance at 440 nm increases when the oxidation of *o*-Tol takes place during the forward scan and decreases in the backward scan, recovering the initial zero value.

CVAs can be used to calculate different thermodynamic parameters of the studied system. Adjusting the sigmoidal shape of the CVA to the Nernst equation,^7,13^ in combination with the Lambert–Beer law (eq Supporting Information 1, Figure S1), the formal potential (*E*^*o*^*′*) of the *o*-Tol/*o*-Tol^+^ redox couple can be calculated, as well as the number of electrons exchanged in the process. This assessment of parameters is fully described in the SI.

Using both the cathodic and anodic scans of the CVA in the parallel configuration, the formal potential, *E*^o^*′* = +0.6960 ± 0.0003 V (*n* = 3), and the number of electrons, *n*_e_ = 1.96 ± 0.01, were obtained. Meanwhile, in the normal configuration, the values of *E*^o^*′* = +0.702 ± 0.002 V (*n* = 3) and *n*_e_ = 1.89 ± 0.02 electrons were calculated. The two results are in good agreement with values reported in the literature^13,16,38,41^ and with the value of *E*^o^*′* obtained from CV, +0.699 V.

The CVA can also provide information about the reversibility of the electrochemical process. Figure S2 shows the derivative voltabsorptograms (DCVAs) at 440 nm in the parallel and normal configurations. As is expected, the derivative of absorbance with respect to time (d*A*/d*t*) exhibited a similar behavior to CV.^16,42^ Analysis of the peak-to-peak difference in DCVA revealed a behavior close to the ideal system in the normal configuration (Δ*Ep*_ox_^red^ = 6 mV); meanwhile, in the parallel configuration, a less reversible behavior is observed (22 mV), due to the contribution of semi-infinite diffusion at the edges of the electrode. More details are provided in the SI.

In summary, the information provided by both CVA and DCVA is consistent with each other, and the obtained parameters of *o*-Tol are in agreement with those reported in the literature, demonstrating the good performance of the Bidim-SEC cell.

### Control of the Thickness of the Thin Layer

In the proposed Bidim-SEC cell, the position of the WE was fixed with a piezoelectric micrometric positioner. This setup provides the possibility of controlling the distance between the WE and the quartz substrate where the optical fibers are fixed, which allows us to perform studies at different distances in a simpler way.

The thickness of the thin layer (*d*) is defined as the distance between the WE and the quartz slide where the optical fibers for the parallel configuration were fixed. This parameter *d* is directly related to the optical path length in the normal configuration setup, and thus changes in *d* lead to clear changes in the response in the normal configuration.

**Figure 3:**
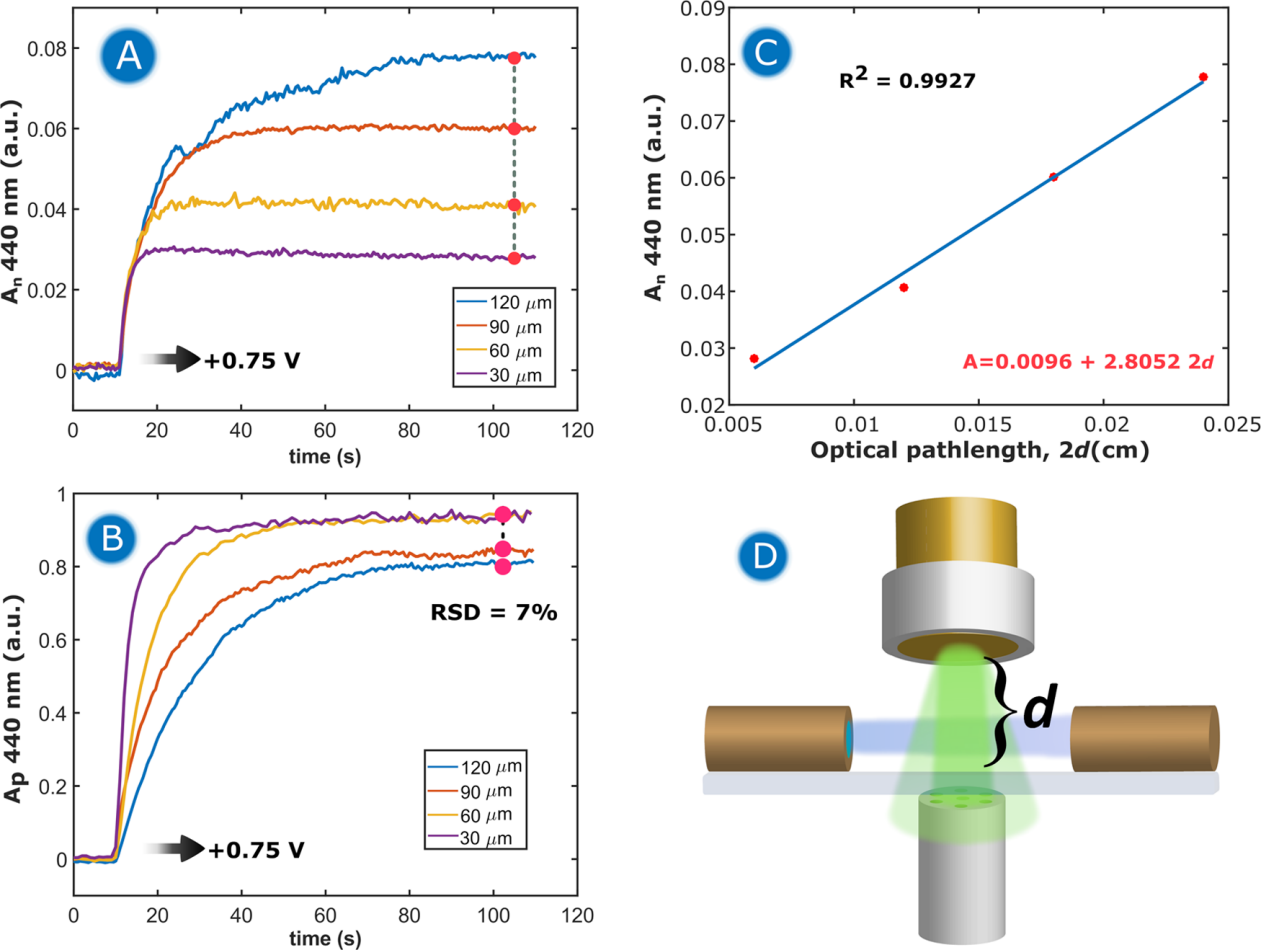
Chronoamperometric oxidation of 0.05 mM *o*-Tol + 1 M HClO_4_ + 0.5 M acetic acid at different thin-layer thickness values. (A) CAbs in the normal configuration at 440 nm for *o*-Tol oxidation. (B) CAbs at 440 nm in the parallel configuration. (C) Correlation between the thin-layer thickness (*d*) and the mean of the absorbance values in the normal configuration at 440 nm between 85 and 105 s. (D) Schematic of the experimental setup, pointing out d, the variable which defines the thin-layer thickness.

Figure 3 shows the results obtained for double pulse chronoamperometry experiments performed at different thicknesses of the thin layer. In these experiments, an initial pulse of +0.50 V was applied for 10 s to set the reference spectrum. Later, a second pulse of +0.75 V was applied for 100 s to promote the oxidation of *o*-Tol to the *o*-Tol^+^ cation. Figure 3A shows the chronoabsorptogram (CAbs) or evolution of the absorbance at 440 nm in the normal configuration with time. As can be observed, as *o*-Tol^+^ was formed, the absorbance increased, reaching a steady value, whose value depends on the thin-layer thickness.

It is to be noted that the time needed to reach a steady value of absorbance at 440 nm due to oxidation of *o*-Tol increased with *d*. Therefore, the maximum absorbance was reached much quicker in the experiments at lower *d* values. In the most extreme case studied, *d* = 120 μm, the absorbance reached a steady value around 75 s after the application of the oxidation pulse. This behavior is related to the time employed to fully electrolyze all of the *o*-Tol present in the thin-layer volume to the *o*-Tol^+^ cation, which depends on the diffusive regime of species in solution.

As expected, the maximum of the absorbance registered in the normal configuration is linearly dependent on the parameter *d*, following the Lambert–Beer law: *A* = ε*Cl*. In the normal configuration, the optical path length (*l*) is defined as 2*d*, two times the thickness of the thin layer, since the experiments were performed in a near-normal reflection mode. Thus, a linear regression between the optical path length and the maximum of absorbance reached in the normal configuration was performed, showing a clear correlation (Figure 3C). The linear fitting can be used to obtain the molar absorptivity coefficient of *o*-Tol, since the slope (2.80 cm^–1^) can be compared with the parameter ε*C* in the Lambert–Beer law, obtaining a value of ε_440_ = 5.6 × 10^4^ M^–1^ cm^–1^, which is in good agreement with the values reported in the literature.^42,43^

A similar behavior is observed in the parallel configuration, where the highest *d* values require more time to reach the total conversion of *o*-Tol to its oxidized form. Figure 3B shows a clear trend between *d* and the time to reach a steady absorbance. The experiments with a higher volume in the thin-layer regime required more time for complete electrolysis of the redox species.

In Figure 3B, it can be observed that the maximum absorbance reached in the parallel configuration exhibits some variation. The origin of this deviation lies in the placement of the WE: although our experimental setup allows us to maintain control over the thickness of the thin layer (the distance between the WE and the quartz slide), the control of the position in the horizontal axis is challenging when a disk electrode is used. This leads to some irreproducibility in the optical pathway sampled in the parallel configuration, due to the circular geometry of the working electrode. The optical fiber is 100 μm in diameter, and the disk is 2.9 mm in diameter. Thus, a small change in the position of the fiber leads to a change in the absorbance values in the parallel arrangement. Nevertheless, the experimental error is only 7 % in these experiments. The origin of this irreproducibility is presented in Figure S3. Although this irreproducibility in the parallel configuration is small, it could be easily avoided by using rectangular electrodes, which would maintain a constant optical path at any position. However, when normalizing the data between 0 and 1, a clearer trend of stabilization time can be observed (Figure S3-A)

### UV/vis Bidimensional SEC Study of Gold Surface Oxidation

Once the new Bidim-SEC cell was validated, we used this setup to study the electrochemical ORC of a Au disk electrode in chloride media. This electrode process is highly relevant in fields such as the electrogeneration of SERS substrates^44^ and numerous catalytic applications.^45−47^ Bidim-SEC provides direct and very valuable information about this complex process in a simple way.

**Figure 4:**
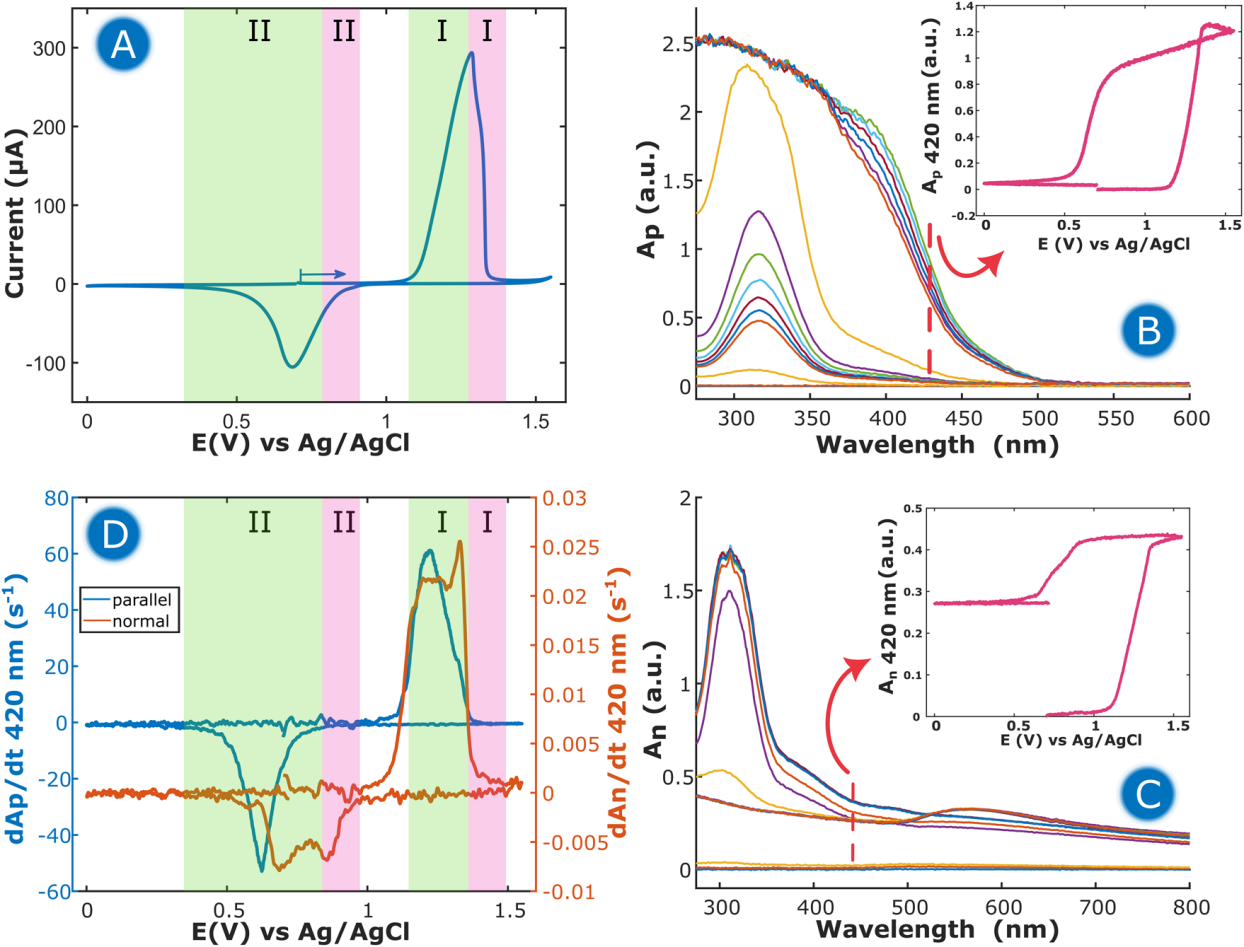
(A) CV response of a Au disk electrode in 0.1 M KCl at 5 mV s^–1^ in the thin-layer regime (120 μm). Evolution of UV/vis absorption spectra obtained in (B) parallel and (C) normal configurations. Insets in (B) and (C) represent CVAs at 420 nm in the corresponding configuration. (D) DCVAs at 420 nm for parallel (blue line) and normal (orange line) configurations.

Figure 4A shows the CV curve, where the evolution of an anodic peak centered at +1.25 V during the forward scan can be observed. This process is related to the electrochemical dissolution of the Au surface to generate AuCl_4_^–^ complexes in solution.^48,49^ This anodic signal drops abruptly around +1.30 V. According to the literature, this behavior is attributed to the formation of an insoluble layer of Au(OH)_3_ and gold oxides, which passivates the electrode surface, hampering the electrochemical dissolution of the Au electrode surface.^50,51^ During the backward scan, the reduction of Au(OH)_3_ and the AuCl_4_^–^ complex around +0.70 V is observed.

More information about the behavior of the electrode–solution interface can be obtained by analyzing the optical signals provided by Bidim-SEC. Figure 4B depicts the evolution of the optical signal recorded in the parallel configuration, where an intense absorption band centered around 320 nm can be observed. This band is related to the generation of the AuCl_4_^–^ complex in solution during the oxidation of the Au electrode surface.^52^ Unfortunately, the amount of AuCl_4_^–^ complex formed during this process is so high that it almost completely absorbs the incident light, resulting in the saturation of the absorbance measurements. Because of this, to study the evolution of the absorbance of AuCl_4_^–^ with respect to the applied potential, a wavelength of 420 nm was selected, where the tail of the absorption band of the complex in solution is observed without saturating the absorbance signal. The CVA in a parallel configuration at 420 nm is presented in the inset of Figure 4B. An abrupt increase of absorbance is observed at +1.10 V, related to the generation of the AuCl_4_^–^ complex, which is reduced at +0.65 V in the backward scan, where a decrease of absorbance to zero is recorded.

In the normal configuration, Figure 4C, other processes with high influence on the optical response are observed. Again, the evolution of an absorption band centered at 320 nm, corresponding to the generation of AuCl_4_^–^ is observed. In this case, due to the shorter optical pathway, a well-defined absorption band was recorded. In the normal configuration, not only the solution adjacent to the electrode but also the electrode surface was interrogated. For this reason, the spectra registered in this configuration are different from those obtained in the parallel configuration. Thus, an absorption band centered at around 570 nm evolved when the potential was scanned in the cathodic direction. Since this band is only observed in the normal configuration, it should be assigned to processes taking place on the electrode surface, generating species that do not diffuse into the solution. This band is ascribed to the generation of AuNPs on the electrode surface during the reduction of the AuCl_4_^–^ complex,^53,54^ showing a typical plasmon band. Also, a remarkable increment of the baseline of the UV/vis absorption spectra in the normal configuration was observed during the oxidation of the gold electrode, which is related to the change in the reflectivity of the surface, due to the formation of Au(OH)_3_ on the electrode surface.^53^

To further provide a deeper insight into the process, additional data treatment can be performed. As aforementioned, a better correlation between absorbance and electrochemical data can be obtained by comparing DCVA and the CV curve, since the shapes of both signals are analogous. Figure 4D shows a representation of the DCVAs at 420 nm in the two configurations.

The DCVA in the parallel configuration (Figure 4D, blue line) provides insights only about the formation of soluble species, in this case, AuCl_4_^–^ which is formed during the electrochemical dissolution of the electrode, from +1.00 to +1.28 V, represented by the green area denoted as I (Figure 4A,D). When a potential of +1.30 V is reached, the formation of Au(OH)_3_ takes place, abruptly dropping the anodic peak in the CV curve (Figure 4A) and changing the whole absorbance of the surface (Figure 4D, pink area denoted as I).

During the backward scan, the CV curve shows a wide cathodic peak that extends from +0.95 to +0.30 V. Bidim-SEC results reveal that there are two processes taking place in this cathodic peak. During the first steps of reduction, the Au(OH)_3_ passivating layer is reduced in two steps, as shown by the decrease in the DCVA in the normal configuration (Figure 4D). Then, the AuCl_4_^–^ complex in solution can be reduced, as is observed both in parallel and normal configurations (Figure 4D, green area denoted as II).

Finally, the reduction of AuCl_4_^–^ leads to the formation of AuNPs on the electrode surface, which are detected by the evolution of a plasmonic band centered at 570 nm, only visible in the normal configuration (Figure 4C) because this thin layer of nanoparticles cannot be detected in the parallel configuration. DCVA in the normal configuration at 570 nm (Figure S4) shows that the evolution of this plasmonic band related to the electrogeneration of AuNPs occurs concomitantly with the reduction of AuCl_4_^–^ after the reduction of insoluble Au(OH)_3_.

In summary, Bidim-SEC has been successfully used to deconvolve the processes taking place during the ORC of the Au disk electrode in KCl medium, demonstrating the generation of AuNPs on the electrode surface during the reduction of AuCl_4_^–^. Additionally, the formation of AuCl_4_^–^ in solution is clearly observed. The electrode is blocked by the generation of a passivation layer of Au(OH)_3_ on the electrode surface at approximately +1.30 V. During the backward scan, the reduction of AuCl_4_^–^ is preceded by the reduction of Au(OH)_3_, denoted by the differences observed between the normal and parallel configuration spectroscopic responses.

## Conclusions

A new Bidim-SEC cell for thin-layer measurements with a controlled thickness was developed. The new device was validated with *o*-Tol, demonstrating the usefulness and versatility of the device to perform SEC experiments in order to obtain valuable information about the molecule or chemical system under study.

This new device was also used to study the ORC of the Au disk electrode in KCl medium. Bidim-SEC measurements allowed us to deconvolve the electrochemical reactions taking place on the electrode surface and in the solution adjacent to it. The generation of soluble AuCl_4_^–^ was observed in parallel and normal configurations during the oxidation of the electrode, whereas the formation of a Au(OH)_3_ layer that passivates the electrode was observed only in the normal configuration. During the backward scan, two reduction processes were observed: (1) the reduction of Au(OH)_3_ and then (2) the reduction of AuCl_4_^–^ to generate AuNPs. These two processes can be clearly observed in the normal configuration both with the CVAs and with the DCVAs, which allowed us to assign unequivocally the processes taking place on the electrode surface, deconvolving the electrochemical signal.

The new experimental setup exhibits a high potential to study complex electrochemical processes in different fields of chemistry, where adsorption and deposition of species take place. A relevant application of the device could be the study of the in situ generation of SERS and other Raman enhancing substrates, which will be addressed in the near future.

## Supporting Information Available

In depth details of the experimental setup, information regarding calculation of the formal potential and exchanged electrons for the *o*-Tol system, DCAs for normal and parallel configurations during *o*-Tol oxidation, schematics regarding the influence of electrode position in the parallel configuration and DVA at 570 nm in the normal configuration for gold oxidation (PDF)
