# Supplementary material for: Bidimensional Spectroelectrochemistry with Tunable Thin-Layer Thickness
Source: Anal Chem. 2024 May 30;96(24):9927–34. doi: 10.1021/acs.analchem.4c01132 (PMC11190879; doi:10.1021/acs.analchem.4c01132)
Supplement: Supplementary file 1 — ac4c01132_si_001.pdf [file ac4c01132_si_001.pdf]

<u>Supporting information</u>

# Bidimensional spectroelectrochemistry with tunable thin-layer thickness

Martin Perez-Estebanez[1], Juan V. Perales-Rondon[1,2], Sheila Hernandez[1,3], Aranzazu Heras[1], Alvaro Colina[1]*

1 Department of Chemistry, Universidad de Burgos, Pza. Misael Bañuelos s/n, E-09001 Burgos, Spain.

2 Hydrogen and Power-to-X Department, Iberian Centre for Research in Energy Storage, Polígono 13, Parcela 31, «El Cuartillo», E-10004 Cáceres, Spain.

3 Chair of Analytical Chemistry II, Faculty of Chemistry and Biochemistry, Ruhr University Bochum, Bochum 44801, Germany.

## Table of contents

Details on experimental section ........ 2

Calculation of formal potential and number of electrons ........ 3

Derivative voltabsorptogram (DCVAs) of o-Tol ........ 4

Influence of working electrode position in parallel configuration ........ 5

DCVA at 570 nm during oxidation-reduction cycles of a Au electrode. ........ 6

## Details on experimental section

***Reagents***

Perchloric acid ($HClO_4$, 60 %, Sigma-Aldrich), lithium perchlorate ($LiClO_4$, >99 % ACROS), o-tolidine (o-Tol, Aldrich, >95 %), glacial acetic acid (Analar, VWR chemicals), potassium chloride (KCl, 99 %, Acros Organics).

All reagents were used as received without further purification. All solutions were prepared using ultrapure water obtained from a Millipore DirectQ purification system provided by Millipore (18.2 M Ω·cm resistivity at 25 °C).

***UV/vis spectroelectrochemistry***

Bidimensional time-resolved UV/vis absorption spectroelectrochemistry was performed using two customized SPELEC instruments (Metrohm-DropSens), each one including a halogen and deuterium light source, a potentiostat and a spectrometer, all controlled by a software (DropView SPELEC software, Metrohm-DropSens), allowing us to synchronize the acquisition of electrochemical and spectroscopic information. Only one light source was used to perform the measurements in both optical configurations, using a bifurcated optical fiber (200 µm, Ocean Optics).

Reflection probe used in normal configuration (DRP-RPROBE, Metrohm-DropSens) consist of a total of seven 200 µm optical fibers: six of them were used to channel the light provided by the source lamp, and one optical fiber was used to collect the light reflected by the WE surface. The thickness of the quartz crystal helps to obtain a good focal distance respect to the position of the WE when the WE is placed between the two optical fibers in parallel arrangement.

*Electrochemistry: validation of cell with o-Tol.*

A cyclic voltammetry at 1 $mVs^{-1}$ was performed to validate the Bidim-SEC cell with 0.05 mM o-Tol + 1 M $HClO_4$ + 0.5 M acetic acid solution, starting at +0.50 V (vs Ag/AgCl 3 M) and reaching the potential vertex at +0.85 V.

*Electrochemistry: study of gold oxidation in KCl*

A cyclic voltammetry in 0.1 M KCl at 20 $mVs^{-1}$ was applied between +1.55 V and 0 V (vs Ag/AgCl 3 M), starting from +0.70 V in the anodic direction to study the oxidation process of a gold WE.

## Calculation of formal potential and number of electrons

Using the Nernst equation, combined with the Lambert-Beer Law, the Equation S1 is obtained.

$$E = E^{0\prime} + 2.3\frac{RT}{n_e F}\log\left(\frac{A_{ox}}{A_{max} - A_{ox}}\right) \quad \text{(ES1)}$$

Parameters in Eq. (ES1) represent: E: measured redox potential, $E^{o\prime}$: formal redox potential, R: gas constant, T: temperature, $n_e$: number of electrons exchanged, F: Faraday constant, $A_{ox}$: absorbance of the oxidized compound as function of applied potential, $A_{max}$: maximum of absorbance after total electrolysis.

Analyzing the values of absorbance at 440 nm, the maximum of absorbance of the *o*-$Tol^{+}$ cation, it is possible to represent the evolution of absorbance versus applied potential, as shown in Figure S1. From this representation, it is possible to obtain the value of $n_e$ and $E^{o\prime}$ from the intercept and slope of the linear regression. The obtained value for $E^{o\prime}$ was then compared with the experimental value of potential observed in the CV, E=0.699. This value was obtained averaging the potential where the maximum current is observed during the oxidation and reduction of *o*-Tol.

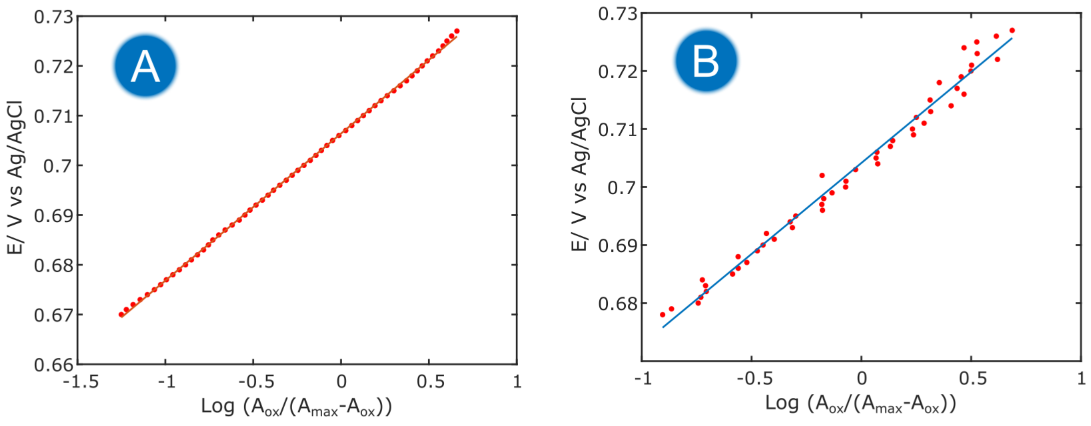


**Figure S1.** Representation of applied potential as a function of $\log(\frac{A_{ox}}{A_{max}-A_{ox}})$ during the oxidation of *o*-Tol for (A) parallel and (B) normal configuration. Experimental conditions were the same as the experiments presented in Figure 1.

## Derivative voltabsorptogram (DCVAs) of *o*-Tol

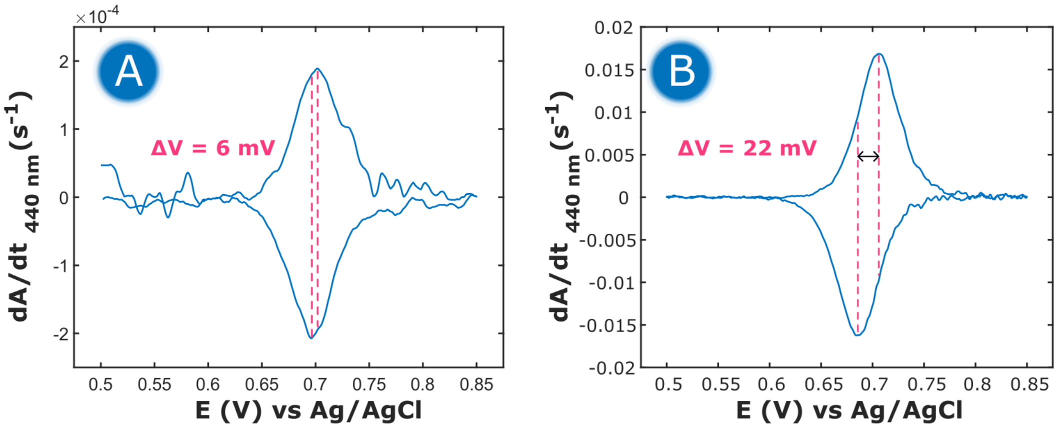


**Figure S2.** DCVAs at 440 nm in (A) normal and (B) parallel configuration. Experimental conditions were the same as used in Figure 2 in the main text.

Figure S2 represents the derivative of the voltabsorptograms (DCVAs) of *o*-Tol shown in Figure 2, in the main text. Analyzing the peak-to-peak difference in Figure S2 reveals a close value to an ideal reversible system ($\Delta E_{ox}^{red} = 0$) in both configurations, obtaining values close to the one observed in the CV. The small divergence in the values could be attributed to the contribution of the edges of the electrode, which is appreciable in parallel but not in normal configuration. Since our experimental setup is not a closed system, some semi-infinite diffusion regime can be found at the edges of the electrode, which shows a higher influence on the electrical signal and on the optical signal in parallel arrangement, because in this optical configuration the solution of the edges is also sampled. Peak to peak separation in the CV and in the DVA in parallel configuration were 23 and 22 mV, respectively. This value is only 6 mV in normal configuration, where the optical signal is sampled at some point close to the geometric center of the electrode, where the concentration profiles of the involved species are more similar to a closed system, and thus the reversibility of the process appears to be better than in the parallel configuration. Nevertheless, the information obtained in the two optical configurations and in the electrochemical signal is very similar for a simple diffusive redox couple, demonstrating the good performance of the new UV/vis-Bidim-SEC cell.

## Influence of working electrode position in parallel configuration

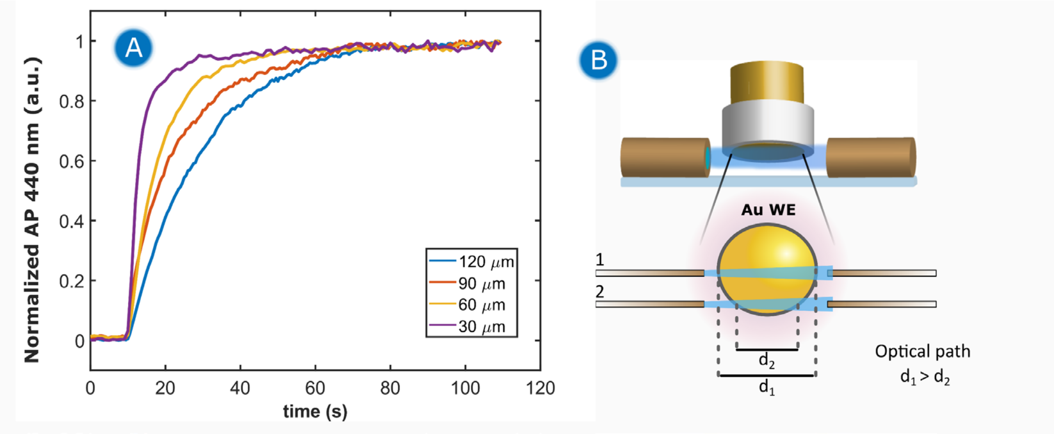


**Figure S3.** (A) Normalized CAbs in parallel configuration at 440 nm. (B) Schematic of the influence of optical fibers position in the optical path of parallel UV/vis-SEC. Experimental conditions as in Figure 3 in the main text.

Figure S3-A shows the normalized absorbance signals in parallel configuration registered during the chronoabsorptometric experiment shown in Figure 3, during the oxidation of o-Tol at +0.75 V. With this representation, it is easier to identify the influence of thin layer thickness on the stabilization time of the absorbance.

In the absorbance raw data, presented in Figure 3-B, a value of irreproducibility in the optical pathway for parallel configuration of around 7 % is calculated. This error is originated by the irreproducibility of the WE positioning, as represented in Figure S3-B, which represents how placing the optical fibers far away from the central point of the electrode results in a lower optical path. Square or rectangular electrodes could be used to avoid this irreproducibility. The different in size of fiber (100 μm diameter) and gold disk electrode (2.9 mm diameter) has to be taken into account, Figure S3B is only illustrative, with the size of the two elements being very different at real scale. In addition to this, the dimension of d1 and d2 parameters have been exaggerated to illustrate the influence of electrode positioning in optical response. In our case, the difference among these values is less than 7% (Figure 3B).

The Stokes-Einstein equation predicts that time required for total electrolysis in thin layer is proportional to the square of the thin-layer thickness. Experimentally, small deviations could be observed due to the fiber location (Figure S3B), making the measurements to exhibit different edge contributions due to the different optical pathways. For this reason, measurements in parallel arrangement should be performed using a well-defined electrode geometry.

## DCVA at 570 nm during oxidation-reduction cycles of a Au electrode.

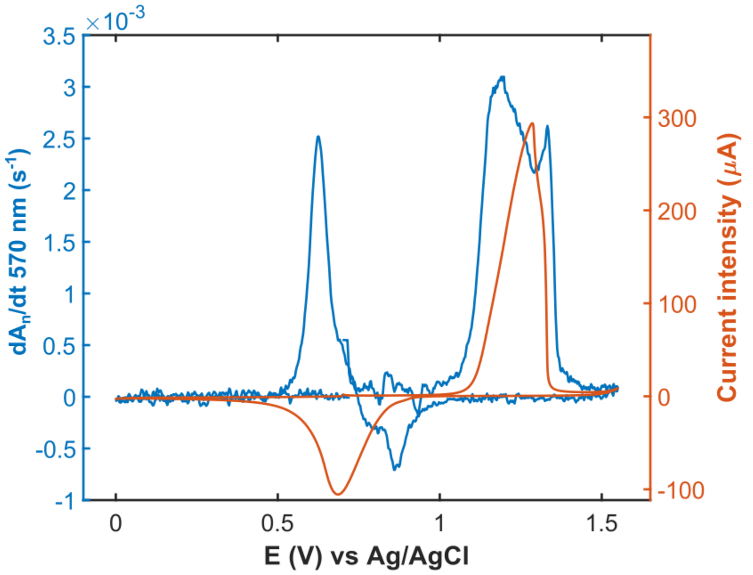


**Figure S4.** DCVA at 570 nm in normal configuration (blue line). This wavelength corresponds to the absorbance of plasmonic bands of AuNPs. CV registered during the experiment (orange line). Experimental conditions as in Figure 4 in the main text.

Figure S4 represents the DCVA in normal configuration at 570 nm during the oxidation-reduction cycle of a Au electrode in 0.1 M KCl. The derivative signal exhibits two clear peaks in the anodic region, during the oxidation of Au WE. We attribute these peaks (+1.15 V and +1.30 V) to the changes in the reflection of the WE caused by the electrochemical dissolution of its surface, to form $AuCl_4^-$ complex (+1.15 V) and also an insoluble layer of $Au(OH)_3$ (+1.30 V). These changes in the reflectivity can be observed in the full UV/Vis normal spectra (inset, Figure 4C) as an increment of the absorbance background signal.

During the cathodic scan, two processes are observed during the WE reduction. The first process, with negative signal, represents the reduction of $Au(OH)_3$. The reduction of the hydroxide leads to an increment of the reflectivity of the surfaces, which translates to lower absorbance. Further in the reduction, the reduction of $AuCl_4^-$ complex is observed around +0.65 V, where noticeable positive signal is observed in the normal DCVA at 570 nm. This signal represents an increment of the absorbance at this wavelength, which is associate to the formation of AuNPs, which exhibit a clear plasmonic band at 570 nm (Figure 4C).
